# Not All Liver Abscesses Are Created Equal: The Impact of Tylosin and Antibiotic Alternatives on Bovine Liver Abscess Microbial Communities and a First Look at Bacteroidetes-Dominated Communities

**DOI:** 10.3389/fmicb.2022.882419

**Published:** 2022-04-27

**Authors:** Lee J. Pinnell, Carla Weissend Whitlow, Katherine L. Huebner, Tony C. Bryant, Jennifer Martin, Keith E. Belk, Paul S. Morley

**Affiliations:** ^1^Veterinary Education, Research, and Outreach Program, Texas A&M University, Canyon, TX, United States; ^2^Department of Animal Sciences, Colorado State University, Fort Collins, CO, United States; ^3^Five River Cattle Feeding, Johnstown, CO, United States

**Keywords:** liver abscess, cattle, microbiome, bovine gut microbiota, Bacteroidetes, *Fusobacterium*

## Abstract

Liver abscesses (LAs) are extremely prevalent in cattle and result in significant economic losses due to liver condemnation, decreased growth and production, and lower carcass quality. LAs are commonly attributed to the transition to diets high in rapidly fermentable starch which results in rumen epithelial inflammation that allows pathogenic bacteria to gain entry to liver through transport *via* the hepatic portal vein. The most common intervention for LAs is the inclusion of antibiotics in feedlot diets, under the supervision of a veterinarian; this treatment is associated with reduced occurrence of LAs in this and other studies. Here, through the largest LA 16S rRNA gene sequencing study to date, we demonstrate that the inclusion of tylosin and antibiotic alternatives (the essential oil limonene and *Saccharomyces cerevisiae* fermentation product) had little impact on LA microbial community composition. Importantly, members of Bacteroidetes (*Bacteroides* spp. and *Porphyromonas* spp.) were identified as the dominant taxa in conjunction with low proportions of Fusobacteria in nearly a quarter (61/259) of all LA communities analyzed in this study. The relative abundances of the phyla Fusobacteria and Bacteroidetes had a strongly negative correlation, and LA microbial communities rarely contained high abundances of both of these dominant phyla. Further, based on the presence of taxa discriminant of Bacteroidetes-dominated LAs within over 400 bovine gut communities, we provide evidence suggestive of Bacteroidetes-dominated abscess communities originating in more distal portions of the bovine gut. Together, these findings suggest that some LA microbial communities may originate from portions of the gut other than the rumen.

## Introduction

Liver abscesses (LAs) are the leading cause of liver condemnation in cattle, and the presence of abscesses resulted in the condemnation of 17.8% of all commercial beef cattle livers in the United States in 2016 (Eastwood et al., 2017). In 2011, the estimated annual loss resulting from abscess condemnations was over $15 million for the United States beef industry alone (Hicks, 2011). In addition to condemnation-based losses, the presence of severe LAs frequently results in decreased cattle production and growth, and lower carcass yield and quality (Brown and Lawrence, 2010; Reinhardt and Hubbert, 2015). For example, the presence of severe LAs can be associated with decreases in average daily weight gain as great as 11% and decreases in feed efficiency as great as 9.7% (Brink et al., 1990). The prevalence of LAs in cattle is generally believed to be linked to feedlot management, and specifically, it is most commonly attributed to the rapid transition to diets high in rapidly fermentable starch (Reinhardt and Hubbert, 2015). It is widely accepted that this change in diet can cause ruminal acidosis that in turn leads to rumen epithelial inflammation, allowing pathogenic bacteria to gain entry to the rumen’s venous drainage and be transported to the liver *via* the hepatic portal vein (Nagaraja et al., 2005). High-starch rations are most commonly fed to feedlot and dairy cattle in North America, and these animals have traditionally been considered to have the highest risk for LAs at harvest. However, national audits performed in the United States and in Canada have repeatedly reported LA prevalences in cattle that are not typically fed high-starch diets (e.g., cull beef cows) that are equivalent to the LA prevalences in feedlot and dairy cattle (BCRC, 2018; Herrick, 2018), which suggests that the etiopathogenesis is likely more complex.

The established etiological theory is that LAs in cattle are predominantly or exclusively caused by leakage of bacteria from the foregut following ruminal acidosis episodes. Because the portal vein collects all venous drainage from the distal esophagus to the cranial rectum, our research group has been investigating the hypothesis that inflammation and bacterial translocation from other segments of the GIT may also be significant in the occurrence of a LAs.

Despite their highly polymicrobial nature (Weinroth et al., 2017; Amachawadi et al., 2021), *Fusobacterium necrophorum* is generally accepted as the primary etiological agent of bovine LAs (Nagaraja et al., 1999a, 2005; Tadepalli et al., 2009), with potential contributions from *Trueperella pyogenes* and perhaps *Salmonella enterica* (Nagaraja et al., 1999a; Amachawadi et al., 2015). The putative significance of *F. necrophorum* in the formation of abscesses is the result of its nearly ubiquitous presence in culture-based studies (Lechtenberg et al., 1988; Tan et al., 1996; Nagaraja et al., 1999a) and its importance in other diseases afflicting cattle such as diphtheria and foot rot (Nagaraja et al., 2005). In general, other bacteria isolated from LAs (e.g., *Bacteroides* spp., *Clostridium* spp., *Enterobacter* spp., *Pasteurella* spp., *Porphyromonas* spp., and *Prevotella* spp.) have not been implicated in LA pathogenesis and are far less frequently isolated. Culture-independent studies employing 16S rRNA gene sequencing or shotgun metagenomics to investigate LA pathogens within the context of their entire microbial communities are rare. To date, three studies using 16S rRNA sequencing have identified Fusobacteria, Bacteroidetes, and Proteobacteria as the most abundant microbial phyla in LAs (Weinroth et al., 2017; Amachawadi et al., 2021; Stotz et al., 2021). Compared to its prevalence in culture-based studies, the increased abundances of Bacteroidetes and Proteobacteria may suggest they play larger roles within LA microbial communities than previously thought.

The most common method used for the prevention and reduction of the effects of LAs in North America is through the inclusion of low doses of antimicrobial drugs in feedlot diets. The two most commonly used drugs for this purpose are tylosin and chlortetracycline (Nagaraja and Lechtenberg, 2007). Supplementing diets with tylosin is currently the most efficacious prevention method (Nagaraja and Lechtenberg, 2007; Reinhardt and Hubbert, 2015), and tylosin must be continuously included in the diet to cattle once introduced (FDA, 2022). Tylosin is a macrolide antibiotic that inhibits peptide elongation during RNA translation (Tenson et al., 2003) and has been shown to exert inhibitory effects on *F. necrophorum* growth (Lechtenberg et al., 1998). In 2011, tylosin was used in over 70% of feedlots with >1,000 animal capacity in the United States (USDA, 2013). However, increasing concerns about antimicrobial resistance have prompted investigations of antibiotic alternatives for the reduction and prevention of LAs. Essential oils (EO) and *Saccharomyces cerevisiae* fermentation products (SCFP) have been proposed as alternatives because of their putative potential to inhibit *F. necrophorum* growth directly or promoting conditions in the rumen that inhibit *F. necrophorum* growth (Nagaraja et al., 1999b; Samii et al., 2016; Wagner et al., 2016). Results from the two 16S rRNA gene sequencing studies investigating the impact of tylosin on LA microbial community structure have been mixed, with one demonstrating no effect (Amachawadi et al., 2021), and the other suggesting tylosin alters LA microbial community composition (Weinroth et al., 2017).

This study utilized 16S rRNA gene sequencing to characterize the diversity and composition of 259 LA microbial communities collected as part of two separate randomized, controlled intervention trials, and more than triples the amount of publicly available 16S rRNA sequence data from LAs in cattle. Importantly, it tests the impact of tylosin and antibiotic alternatives on LA microbial community structure, and for the first time investigates LAs not dominated by *Fusobacterium*. Further, through a meta-analysis incorporating 422 bovine gut samples, it examines possible origins of LA microbial communities from more distal portions of the bovine GI tract than the rumen.

## Materials and Methods

### Sample Populations and Experimental Design

This study was comprised of two separate blinded, randomized, and controlled trials of steers conducted at large commercial beef feedlots, one in Colorado and another in Texas. The feedlots and rearing conditions were typical of large North American feedlots with >5,000 animal capacity. Complete details about the sample population, cattle handling, base diets, and experimental design of both trials have been described previously (Weissend, 2018; Huebner et al., 2019).

Briefly, in the Colorado-based trial, steers (*n* = 4,689 cattle housed in 28 pens) were enrolled in a randomized, block-controlled study at a commercial feedlot in Northern Colorado. Upon arrival cattle were administered injectable and oral anti-parasitic treatment (Noromectin, Norbook Laboratories, Newry, United Kingdom; Synanthic, Boehringer Ingelheim, Ingelheim am Rhein, Germany; Standguard, Elanco Animal Health, Greenfield, IN, United States) and two injectable bacterin-toxoid vaccines protecting again BRD (Titanium 5 + PH-M, Elanco Animal Health; Vision 7, Intervet/Merck Animal Health, Omaha, NE, United States) and randomly assigned into two adjacent pens based on equal weight distribution. Pens were open-air, dirt-floor with a central feed alley. All cattle received base diets consisting predominantly of steam flaked corn, and cattle in adjacent pens were randomly assigned to receive either (1) a no treatment diet containing no additives for prevention of LAs (negative control) or (2) a diet supplemented with *Saccharomyces cerevisiae* fermentation product (SCFP; 18 g·d^−1^; Diamond V, Cedar Rapids, IA, United States). A treatment and control pen designated one block, and each block contained cattle with similar source locations, bread, headcounts, and weight distributions.

In the Texas-based trial, steers (*n* = 5,481 housed in 40 pens) were enrolled in a randomized, block-controlled study at a commercial feedlot located in West Texas. Following arrival, cattle received a standard processing regimen consisting of drench dewormer, IBR and BVD vaccines, lot tags, and ear implants (Revalor-XS, Merck Animal Health, Madison, NJ, United States). Cattle were then randomly assigned to pens within randomization blocks; each block consisted of four pens with an equal number of cattle, and each pen was randomly assigned one of the four treatments groups. All cattle were provided with corn-based rations *ad libitum* with one of the following four modifications: (1) a no treatment diet containing no additives for prevention of LAs (negative control), (2) a diet supplemented with tylosin (90 mg·d^−1^; Elanco Animal Health), (3) a diet supplemented with the essential oil limonene (1 g·d^−1^; Victus-LIV; manufactured by Prinova Flavors for DSM Nutritional Products), or (4) a diet supplemented with SCFP (18 g·d^−1^; Diamond V). Additionally, cattle in all four treatment groups received monensin (Rumensin, Elanco Animal Health) in their intermediate and finishing rations and ractopamine (Optaflexx, Elanco Animal Health) during the 14 days prior to harvest.

Methods involving animal care and use in both trials were approved by and carried out in accordance with the Colorado State University Research Integrity and Compliance Review Office (Protocol number 102616).

### Sample Collection

At the end of both feeding trials, all livers from cattle were evaluated at the time of harvest for the presence and severity of LAs. The prevalences of A and A+ abscesses within each pen were recorded, and pens were subsequently categorized according to tertiles of the prevalence distribution (i.e., highest-, middle-, and lowest-third of the prevalence values). Up to 5 abscessed livers per pen (*n* ≤ 5 per pen) were selected for sampling. The LAs and surrounding tissue were excised, and this tissue block was placed into sterile sample bags, placed on ice, and transported to the Center for Meat Safety and Quality Food Safety Microbiology Lab at Colorado State University where they were processed within 24 h.

### DNA Isolation, 16S rRNA Library Preparation, and Sequencing

To facilitate aseptic recovery of purulent material, individual tissue blocks containing abscesses (*n* = 259) were immersed in 100% ethanol and then flamed to sterilize the outer surfaces. Abscesses were then aseptically dissected to access the purulent material, and 0.1–0.4 g was harvested. Genomic DNA was isolated using the QIAamp PowerFecal DNA Isolation kit (Qiagen, Hilden, Germany), according to the manufacturer’s instructions. Following isolation, DNA was quantified (ng μl^−1^) using a Qubit flex fluorometer (Thermo Fisher Scientific, Waltham, MA, United States).

16S rRNA gene amplicon library preparation and sequencing were carried out by Novogene Corporation Inc. (Chula Vista, CA, United States). The V4 region of the 16S rRNA gene was amplified using the 515f (5′-GTG CCA GCM GCC GCG GTA A-3′) and 806r (5′-GCA CTA CHV GGG TWT CTA AT-3′) primer pair and Novogene’s proprietary amplification conditions. Following successful PCR, amplicon libraries were prepared and pooled using Novogene’s proprietary process and sequenced on an Illumina HiSeq 2,500 instrument (Illumina Inc., San Diego, CA, United States) using 2 × 250 bp paired-end chemistry. As negative controls, equal volumes of nuclease-free sterile water were included with each batch of samples processed. These controls were included in procedures for PCR amplification and in preparation of sequencing libraries. Sequencing of these negative control samples did not yield product and therefore were not included in further downstream analysis. The number of reads per liver abscess sample ranged from 14, 966 to 973, 255, with an average of 286, 429 reads per sample and there were no differences in read depth between any of the treatment groups (pairwise Wilcoxon rank-sum with Benjamini–Hochberg correction, *p* > 0.05).

### Bioinformatics

Demultiplexed paired-end reads were imported into QIIME2 version 2020.11 (Bolyen et al., 2019) and DADA2 (Callahan et al., 2016) was used to filter reads for quality, remove chimeric sequences, merge overlapping paired-end reads, and generate amplicon sequence variants (ASVs). Forward and reverse reads were trimmed at 25 and 26 bp and truncated at 244 and 249 bp, respectively. Taxonomy was assigned using a Naïve Bayes classifier trained on the Greengenes version 13_8 99% OTUs database (DeSantis et al., 2006), where sequences had been trimmed to include only the base pairs from the V4 region bound by the 515f/806r primer pair. Reads that mapped to chloroplast and mitochondrial sequences were filtered from the representative sequences and ASV table using the “filter_taxa” function, and a midpoint-rooted phylogenetic tree was then generated using the “q2-phylogeny” pipeline with default settings, which was used to calculate phylogeny-based diversity metrics. The proportion of ASVs classified at each taxonomic rank can be found in Supplementary Table S1.

Data and metadata were then imported into phyloseq (McMurdie and Holmes, 2013). Richness (Observed ASVs) and Faith’s Phylogenetic Diversity (FPD) were calculated for all samples with phyloseq and the btools package. ASV counts for each sample were then normalized using cumulative sum scaling (Paulson et al., 2013) and beta-diversity was analyzed using generalized UniFrac distances (Lozupone et al., 2011; Chen et al., 2012). From these distances, non-metric multidimensional scaling (NMDS) was performed and plotted, and a permutational multivariate analysis of variance (PERMANOVA) was used to test for significant differences in community structure using the vegan (Oksanen et al., 2019) and pairwiseAdonis (Arbizu, 2017) packages. To ensure significant differences were not the result of unequal dispersions of variability, permutational analyses of dispersion (PERMDISP) were conducted for all significant PERMANOVA outcomes using the vegan package. Hierarchal clustering was performed using Ward’s agglomeration clustering method (Murtagh and Legendre, 2014) on generalized UniFrac distances and the “hclust” function. Further, the relative abundances of ASVs within each sample were calculated and plotted using phyloseq.

### Fusobacteria and Bacteroidetes-Dominated Communities and Their Discriminant Taxa

To further explore the dichotomy between liver abscess microbial communities dominated by Fusobacteria or Bacteroidetes, samples were classified as belonging to 1 of 5 groups: high Fusobacteria (Fusobacteria relative abundance [RA] > 75%, *n* = 180), high Bacteroidetes (Bacteroidetes RA > 14%, *n* = 61), high Proteobacteria (Proteobacteria RA > 15%, *n* = 9), high Firmicutes (Firmicutes RA > 11%, *n* = 5), or other (*n* = 4). Group cutoffs were set based on relative abundance values that resulted in the least number of samples cross-appointed to two groups (i.e., Fusobacteria >75% and Bacteroidetes >14%). Though largely mutually exclusive, samples (*n* = 3) cross-appointed between high Fusobacteria and high Bacteroidetes were classified as high Fusobacteria and samples (*n* = 3) cross-appointed between high Bacteroidetes and high Proteobacteria were classified as high Bacteroidetes. All other samples (*n* = 253) were mutually exclusive.

To identify the higher-level taxa discriminating Fusobacteria-dominated abscesses (*n* = 180) from Bacteroidetes (*n* = 61) dominated abscesses, linear discriminant analysis effect size (LEfSe) was performed using the online LEfSe tool on the Galaxy server^1^ under default settings, except for using a stricter alpha value and higher logarithmic LDA score threshold (0.01 and 3.0, respectively). Genera making up greater than 0.05% of the overall community across each community type (i.e., high Bacteroidetes or high Fusobacteria) and present in at least 20% off all LA communities were considered.

### Similarities to Microbial Communities From Different GI Tract Components

To identify potential anatomic sources for bacterial communities that were translocated from the GIT to seed the liver, the relative abundances of genera discriminant of both Fusobacteria and Bacteroidetes-dominated abscesses were visualized and compared to bacterial communities of nine GIT locations from a total 56 cattle, using 16S rRNA data from previous research that was publicly available at the NCBI’s Sequence Read Archive (SRA) through BioProjects PRJEB28377 and PRJNA399771. PRJEB28377 represents the largest collection of 16S rRNA sequence data covering multiple GIT locations publicly available at the time of writing and represents nine different segments (rumen, omasum, abomasum, duodenum, jejunum, ileum, caecum, colon, and rectum) of the GIT in healthy cattle and cattle with hemorrhagic bowel syndrome. For the purposes of this study, only samples collected from healthy cattle were included (*n* = 373). Similarly, PRJNA39971 sampled four different segments (rumen, jejunum, caecum, and colon) of dairy calves. All samples collected from this study were included (*n* = 49) here. Demultiplexed sequence reads from both studies (*n* = 422) were downloaded from the SRA and processed as described above in the Bioinformatics subsection.

### Statistical Analyses

Unless otherwise stated, R version 3.6.3 (R Core Team, 2017) was used for statistical analysis of data. The impact of dietary inclusion of tylosin and antibiotic alternatives on microbial community composition were analyzed independently for the Colorado and Texas trials, because of potential confounding related to location and cattle source, and because monensin and ractopamine were only included in the diets of cattle enrolled in the Texas trial. Pairwise Wilcoxon rank-sum tests were performed with a Benjamini–Hochberg correction for multiple comparisons. Differences in beta-diversity were tested using pairwise PERMANOVA with a Benjamini–Hochberg correction for multiple comparisons and 9,999 permutations. Additionally, pairwise PERMDISPs were carried out for all significant PERMANOVA outcomes using 9,999 permutations to test for differences in the variability of dispersions. Correlation between the relative abundance of Fusobacteria and Bacteroidetes was tested using Spearman’s rank correlation coefficient.

### Data Availability

All sequence reads were made available through BioProjects PRJNA472428 (Colorado trial) and PRJNA804092 (Texas trial) at the NCBI’s Sequence Read Archive.

## Results

### Impact of Tylosin, Essential Oil, and SCFP on Liver Abscess Microbial Communities

**Figure 1:**
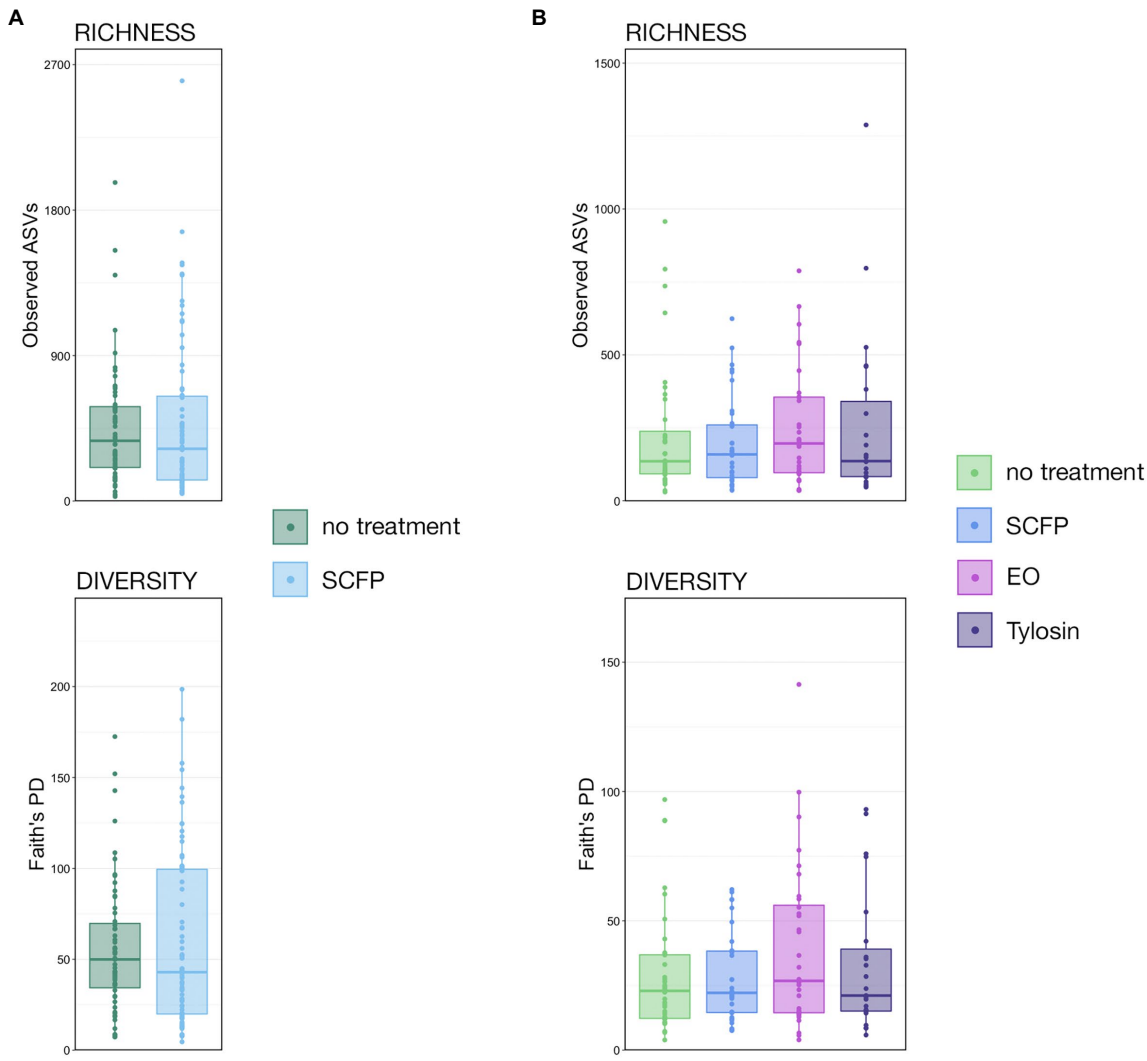
Boxplots demonstrating the numbers of observed amplicon sequence variants (ASVs; richness) and Faith’s phylogenetic diversity between treatment groups in **(A)** the Colorado trial and **(B)** the Texas Trial. No significant differences were detected in the Colorado trial (Pairwise Wilcoxon rank-sum with Benjamini–Hochberg correction, *p* > 0.05, *n* = 59–75) or Texas trial (Pairwise Wilcoxon rank-sum with Benjamini–Hochberg correction, *p* > 0.05, *n* = 23–36).

Previous analyses using the same trials as this study demonstrated that diets supplemented with tylosin decreased the prevalence of LAs (Weissend, 2018), but supplementation with SCFP and EO had no impact on LA prevalence (Huebner et al., 2019). In the Colorado trial, there were no significant differences in the richness or diversity within liver abscess microbial communities from cattle given the control diet (*n* = 59) or the diet supplemented with SCFP (*n* = 74; Figure 1A; pairwise Wilcoxon rank-sum with Benjamini–Hochberg correction; *n* = 59–74, *p* > 0.05). Similarly, there were no significant differences in the richness or diversity of liver abscess communities between cattle given the control diet (*n* = 36), diet supplemented with SCFP (*n* = 35), diet supplemented with essential oil (EO; *n* = 32), or diet supplemented with tylosin (*n* = 23) during the Texas trial (Figure 1B; pairwise Wilcoxon rank-sum with Benjamini–Hochberg correction; *n* = 23–36, *p* > 0.05).

**Figure 2:**
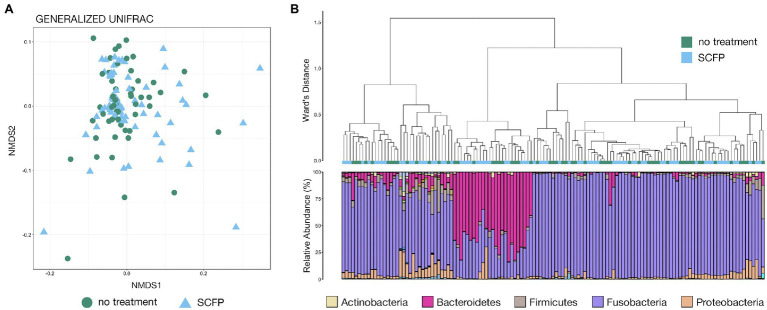
**(A)** Non-metric multidimensional scaling (NMDS) of generalized UniFrac distances illustrating differences in microbial community structure between treatment groups in the Colorado trial. The NMDS demonstrates clustering of 16S rRNA gene sequences from liver abscess (LA) microbial communities from animals given the control diet or diet supplemented with *Saccharomyces cerevisiae* fermentation product (SCFP). **(B)** The relatedness of LA microbial communities from the Colorado trial based on normalized ASVs. Hierarchal clustering was performed on generalized UniFrac distances using Ward’s agglomeration method. Green boxes represent LA communities from animals fed the control diet, and blue boxes represent LA communities from animals given the SCFP-supplemented diet. The bar plot illustrates the relative abundance of microbial phyla within each individual sample. The five most abundant phyla across all samples are displayed in the legend.

**Figure 3:**
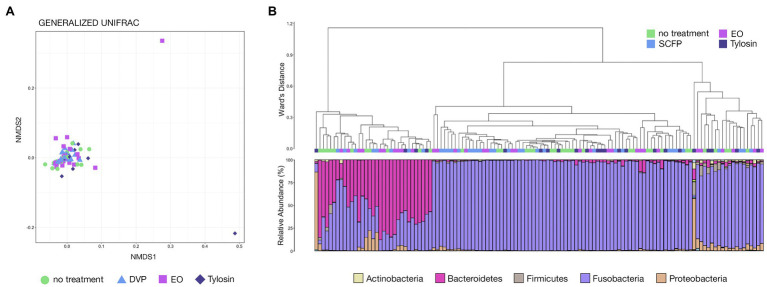
**(A)** Non-metric multidimensional scaling of generalized UniFrac distances illustrating differences in microbial community structure between treatment groups in the Texas trial. The NMDS demonstrates clustering of 16S rRNA gene sequences from LA microbial communities from animals given the control diet, and diet supplemented with SCFP, essential oil (EO), or tylosin. **(B)** The relatedness of LA microbial communities from the Texas trial based on normalized ASVs. Hierarchal clustering was performed on generalized UniFrac distances using Ward’s agglomeration method. Green boxes represent LA communities from animals fed the control diet, blue boxes represent LA communities from animals given SCFP-supplemented diet, light purple boxes represent LA communities from animals given EO-supplemented diet, and dark purple boxes represent LA communities from animals given tylosin-supplemented diet. The bar plot illustrates the relative abundance of microbial phyla within each individual sample. The five most abundant phyla across all samples are displayed in the legend.

Based on generalized UniFrac distances, there were no significant differences in microbial community composition between cattle fed the control diet versus those receiving SCFP-supplemented diet in the Colorado trial and NMDS illustrated no clustering of abscess microbial communities collected from cattle given each diet (Figure 2A; PERMANOVA, *n* = 59–74, *p* > 0.05). There were also no significant differences in abscess-associated microbial community structure between cattle fed the control diet and diet supplemented with SCFP, EO, or tylosin in the Texas trial and NMDS showed no clustering of communities by treatment (Figure 3A; PERMANOVA, *n* = 23–36, *p* > 0.05).

Hierarchal clustering further revealed that treatment (diet supplemented with SCFP) within the Colorado trial had little effect on abscess microbial community structure, as communities from cattle given different diets were intermixed across all clades (Figure 2B). Visualization of microbial community structure at the taxonomic rank of phyla demonstrated that members of Fusobacteria, Bacteroidetes, Proteobacteria, and Firmicutes comprised most of the microbial community, representing over 90% of all microbial taxa in 128 of 133 samples (~96%; Figure 2B). However, the ranges of relative abundances for dominant phyla were extremely large: Fusobacteria, 14.8%–99.3%; Bacteroidetes, 0.02%–82.0%; Proteobacteria, 0.2%–30.0%; and Firmicutes (0.08%–31.3%). Some communities were almost exclusively comprised of Fusobacteria while others are largely composed of Bacteroidetes. Interestingly, clades appeared to form based largely on the abundance of members Fusobacteria and Bacteroidetes, with communities dominated by the latter forming their own distinct clade (Figure 2B).

Within the Texas trial, hierarchal clustering demonstrated that treatment (diet supplemented with SCFP, EO, or tylosin) had little effect on the microbial community structure of LAs, as abscess communities from cattle receiving the different diets were intermixed across all clades (Figure 3B). Visualization of the microbial community composition at the rank of phyla illustrated that members of the phyla Fusobacteria, Bacteroidetes, Proteobacteria, and Firmicutes comprised almost the entirety of the microbial community, representing over 90% of all microbial taxa in 125 of 126 samples (~99%; Figure 3B). Once again, these four phyla exhibited extremely variable ranges of abundance among the individual abscess communities (Fusobacteria, 2.0%–99.8%; Bacteroidetes, 0.03%–88.0%; Proteobacteria, 0.02%–85.1%; and Firmicutes, 0.01%–14.4%). As was the case in the Colorado trial, the formation of clades appeared to be largely influenced by the abundance of Fusobacteria and Bacteroidetes, with Bacteroidetes-dominated communities forming their own distinct clade (Figure 3B).

### Establishing Bacteroidetes as the Dominant Taxa in Some Liver Abscess Microbial Communities

Of the 259 LA microbial communities, the vast majority (241, 93.1%) was dominated by either Fusobacteria (180, 69.4%) or Bacteroidetes (61, 23.6%). The remaining 18 LA communities were classified as high Proteobacteria (9, 3.5%), Firmicutes (5, 1.9%), or other (4, 1.5%). The proportions of LAs with communities considered high Fusobacteria or high Bacteroidetes were similar between the two trials, while communities not dominated by Fusobacteria or Bacteroidetes were far more abundant in the Colorado trial. Importantly, while there was a slightly higher proportion of Fusobacteria-dominated abscess communities in pens with higher liver abscess prevalence, the difference was not statistically significant (Supplementary Figure S1; pairwise Wilcoxon rank-sum with Benjamini–Hochberg correction; *n* = 21–22, *p* > 0.05). Conversely, there was a slightly higher proportion of Bacteroidetes-dominated abscess communities in pens with lower liver abscess prevalence, but the difference was not statistically significant (Supplementary Figure S1; pairwise Wilcoxon rank-sum with Benjamini–Hochberg correction; *n* = 21–22, *p* > 0.05). There were no significant differences in the proportions of Fusobacteria or Bacteroidetes-dominated liver abscess communities between any of the treatment groups, though there was a slightly lower proportion of Bacteroidetes-dominated communities in cattle given tylosin (Supplementary Figure S2; pairwise Wilcoxon rank-sum with Benjamini–Hochberg correction; *n* = 9–24, *p* > 0.05).

**Figure 4:**
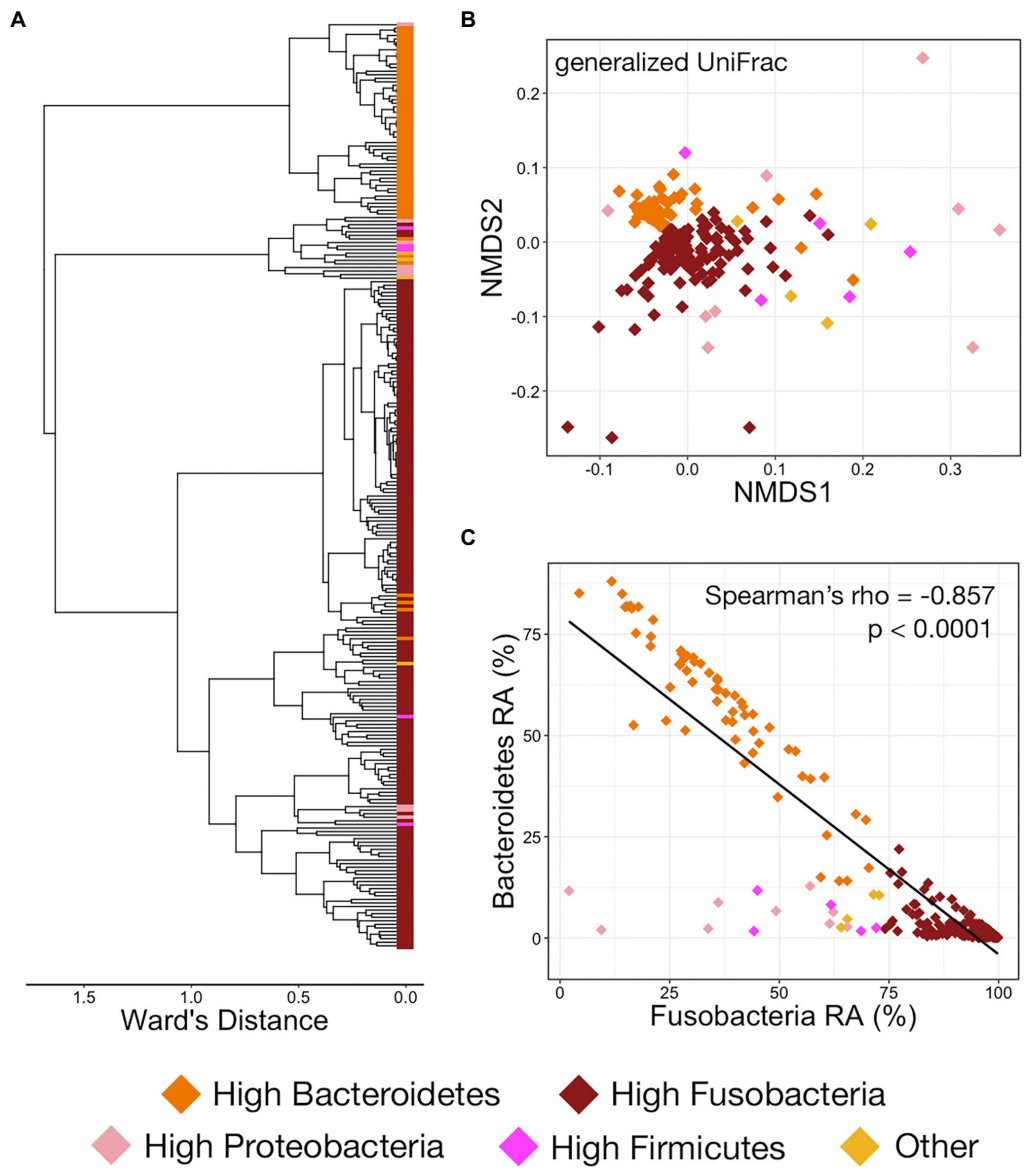
**(A)** Hierarchal clustering using Ward’s agglomeration method of LA microbial communities from both trials illustrating the relatedness of the communities classified as high Bacteroidetes (orange boxes), high Fusobacteria (dark red boxes), high Proteobacteria (light pink boxes), high Firmicutes (magenta boxes), or other (yellow boxes). **(B)** NMDS of generalized UniFrac distances illustrating differences in LA microbial community structure between the five different LA community types. The NMDS demonstrates clustering of 16S rRNA gene sequences from LA microbial communities classified as high Bacteroidetes, high Fusobacteria, high Proteobacteria, high Firmicutes, or other across both trials. **(C)** Scatterplot demonstrating the relationship between the relative abundances of Fusobacteria and Bacteroidetes. Communities are colored based on their assigned community type (high Bacteroidetes, high Fusobacteria, high Proteobacteria, high Firmicutes, or other).

Hierarchal clustering of LAs from both trials showed that the predominance of Fusobacteria or Bacteroidetes was the most important factor in differentiating between abscess microbial communities, with virtually every community dominated by Bacteroidetes forming its own clade that was most distinct from all other clades (Figure 4A). Communities not dominated by Bacteroidetes of Fusobacteria (high Proteobacteria, high Firmicutes, or other) largely clustered with themselves inside of a larger clade comprised of Fusobacteria-dominated communities (Figure 4A). Visualization with NMDS clearly illustrates that communities with high Fusobacteria and high Bacteroidetes both form distinct clusters with communities of their own type, while those with high Proteobacteria, Firmicutes, or other are much more highly variable and do not form distinct clusters (Figure 4B). Both Fusobacteria and Bacteroidetes-dominated communities were significantly different from all other community types, even when only partially weighting taxa abundance (Supplementary Table S2; PERMANOVA, *n* = 61–180, *p* < 0.05). However, the other community types (high Proteobacteria, Firmicutes, or other) were not significantly different from one another (Supplementary Table S2; PERMANOVA, *n* = 4–9, *p* > 0.05). Communities rarely contained high abundances of both Fusobacteria and Bacteroidetes, and their relative abundances within liver abscess microbial communities were strongly negatively correlated (Figure 4C; Spearman’s rho = −0.857, *n* = 259, *p* < 0.0001).

Fusobacteria, and the genus *Fusobacterium* specifically, was the most abundant taxa in LAs considered high Proteobacteria or high Firmicutes but was in lower proportion than LAs classified as high Fusobacteria using criteria described for this analysis (Supplementary Table S3). The relative abundance of other community members within these community types was highly variable. In high Proteobacteria LAs, the genera *Halomonas* (2.9% ± 1.53 SEM), *Agrobacterium* (6.6% ± 6.56 SEM), and unclassified Enterobacteriaceae (2.1% ± 1.53 SEM) were the most abundant members of Proteobacteria (Supplementary Table S3). However, the high relative abundance of *Agrobacterium* was the result of it dominating (59%) a single LA and it comprised less than 0.2% of communities in the other high Proteobacteria LAs. The increased abundance of Firmicutes in LAs considered high Firmicutes was largely the result of unclassified Veillonellaceae (5.5% ± 1.33 SEM), unclassified Ruminococcaceae (2.8% ± 1.18 SEM), and unclassified Clostridiales (1.8% ± 0.30 SEM), which were all among the top five most abundant taxa at the level of genus (Supplementary Table S3).

**Figure 5:**
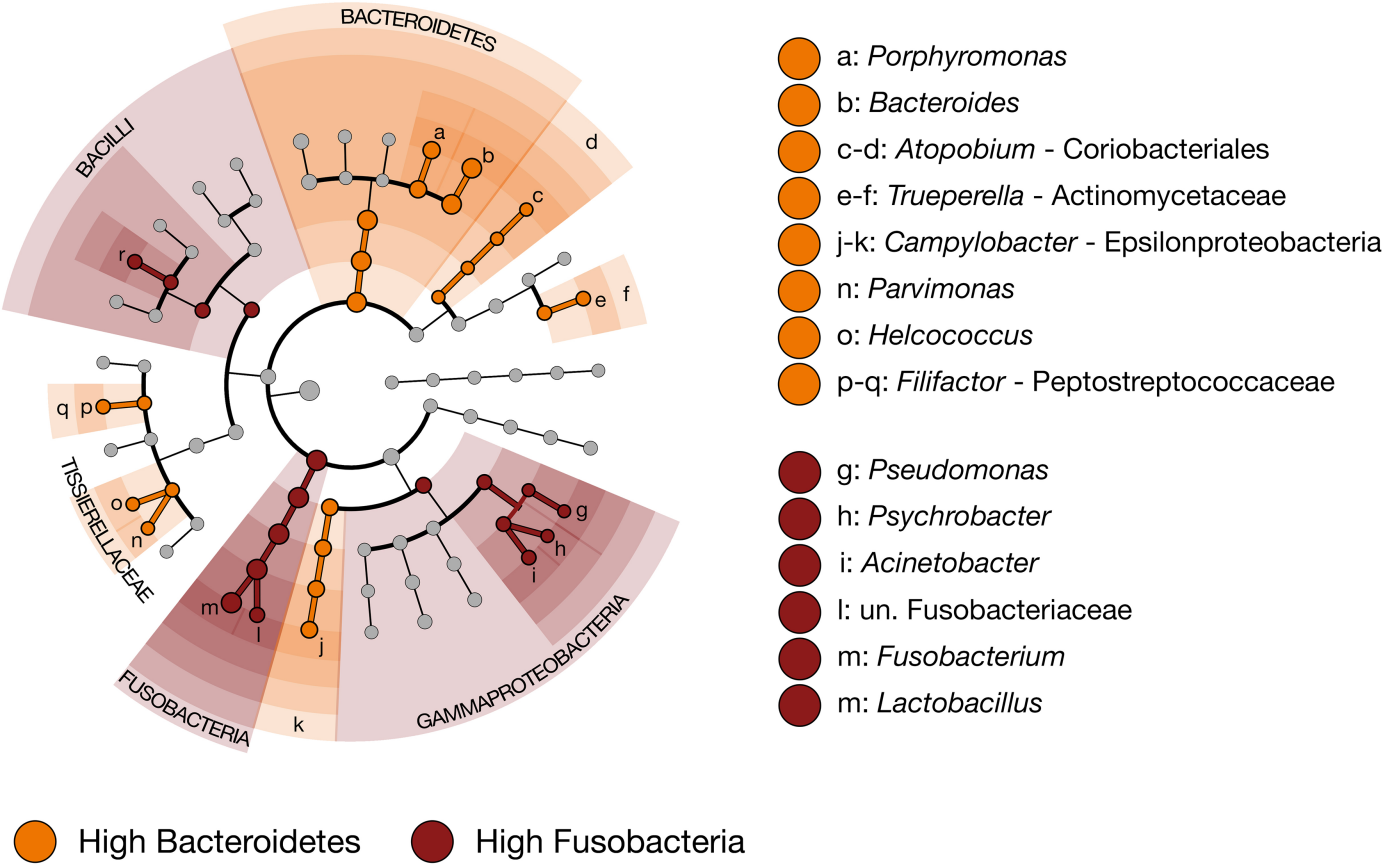
Cladogram demonstrating the microbial taxa with relative abundances of greater than 0.1% of the total community in LA microbial communities classified as high Bacteroidetes (*n* = 61) or high Fusobacteria (*n* = 180). Taxa discriminant of LA communities classified as high Bacteroidetes are highlighted in orange, and taxa discriminant of LA communities classified as high Fusobacteria are highlighted in dark red. (LEfSe, *p* < 0.01, *n* = 61–180).

In addition to *Fusobacterium*, taxa discriminant of Fusobacteria-dominated liver abscess microbial communities included the genera *Acinetobacter*, *Lactobacillus*, *Pseudomonas,* and *Psychrobacter* (Figure 5; LEfSe, *n* = 61–180, *p* < 0.01). Of these genera, *Fusobacterium* was unsurprisingly the most abundant, comprising 92.2% ± 0.48 SEM of the overall community in Fusobacteria-dominated abscesses. The other four discriminant taxa comprised only 0.1%–0.3% of the overall community (Supplementary Table S3). Two genera of Bacteroidetes, *Bacteroides* and *Porphyromonas*, along with the genera *Atopobium*, *Campylobacter*, *Filifactor*, *Helcococcus*, *Parvimonas*, and *Trueperella* were discriminant of Bacteroidetes-dominated liver abscess microbial communities (Figure 5; LefSe, *n* = 61–180, *p* < 0.01). Not surprisingly, *Bacteroides* was the most abundant of the three, representing 51.3% ± 2.67 SEM of the overall community in Bacteroidetes-dominated LA communities. *Porphorymonas* (4.9% ± 1.37 SEM) and *Campylobacter* (2.6% ± 0.78 SEM) were moderately abundant, while the other six discriminant taxa comprise 0.1%–0.3% of the overall community (Supplementary Table S3). The genus *Salmonella* was not identified in any sample.

### Taxa Discriminant of Fusobacteria and Bacteroidetes-Dominated Liver Abscesses and Their Potential Origin in the Bovine Gut

**Figure 6:**
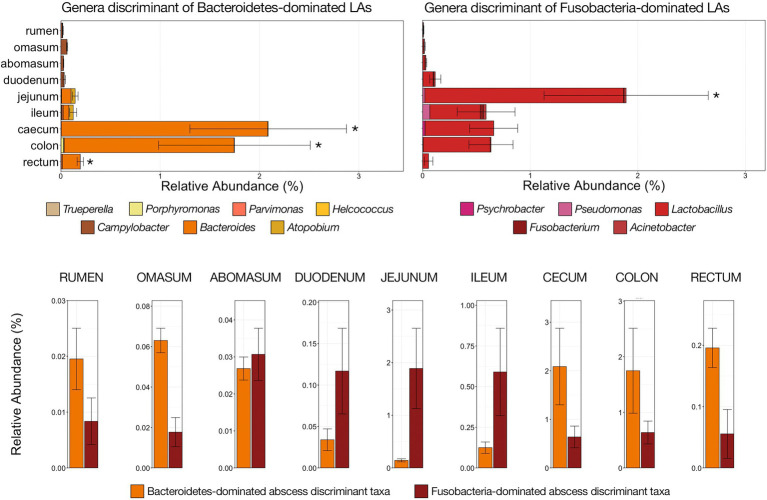
Bar plots demonstrating the relative abundances of all genera discriminant of LA microbial communities classified as high Bacteroidetes (left; shades of orange) and high Fusobacteria (right; shades of red) with nine locations of the bovine gastrointestinal tract (GIT). All genera detected are listed in the legends. Error bars represent the SEM. Significant differences between GIT locations are noted by an asterisk for the aggregate relative abundance of discriminant genera of high Bacteroidetes communities (Pairwise Wilcoxon rank-sum with Benjamini–Hochberg correction, *p* < 0.05, *n* = 34–56) and high Fusobacteria communities (Pairwise Wilcoxon rank-sum with Benjamini–Hochberg correction, *p* < 0.05, *n* = 34–56). Bar plot demonstrating the aggregate abundance of genera discriminant of high Bacteroidetes or high Fusobacteria LA communities at each of the nine GIT locations investigated sorted from left (proximal GIT) to right (distal GIT). Error bars represent the SEM.

Genera discriminant of the community composition types assigned to LAs were similarly investigated in 422 samples collected from nine locations within the bovine GIT in two previous studies (rumen, omasum, abomasum, duodenum, jejunum, ileum, caecum, colon, and rectum). In general, discriminant taxa that were abundant in LAs (i.e., *Fusobacterium*, *Bacteroides*, and *Porphorymonas*) were considerably less abundant in the GI tract. In aggregate, taxa discriminant of Fusobacteria-dominated abscesses were significantly more abundant within the jejunum than all other GIT locations while taxa discriminant of Bacteroidetes-dominated abscesses were significantly more abundant within the cecum, colon, and rectum than all other locations (Figure 6; pairwise Wilcoxon rank-sum with Benjamini–Hochberg correction; *n* = 34–88, *p* < 0.05). Within the rumen, omasum, and abomasum, the relative abundance of both sets of discriminant taxa were incredibly low (<0.08%). However, within more proximal portions of the GIT (i.e., abomasum, duodenum, jejunum, and ileum), taxa discriminant of Fusobacteria-dominated abscess communities were more abundant than those discriminant of Bacteroidetes-dominated abscesses (Figure 6). Conversely, genera discriminant of Bacteroidetes-dominated LAs were more abundant than taxa discriminant of Fusobacteria-dominated LAs in the cecum, colon, and rectum (Figure 6).

## Discussion

This study used 16S rRNA gene sequencing to investigate how dietary inclusion of tylosin (the most common intervention used to reduce/prevent LAs) and antibiotic alternatives (essential oil—EO and *S. cerevisiae* fermentation products—SCFP) impacted the microbial community structure of LAs in cattle. Results suggest that diet supplementation with tylosin, EO, or SCFP had little impact on the richness, diversity, or composition of LA microbial communities. Previous research utilizing the same trials as this study showed that cattle receiving diet supplemented with tylosin were significantly less likely to develop LAs, but cattle receiving diets with EO or SCFP were just as likely to develop abscesses as the control group (Weissend, 2018; Huebner et al., 2019), which corroborates the findings of other studies regarding the impact of tylosin as a preventive intervention for LAs (Reinhardt and Hubbert, 2015). More importantly, this study also identified members of Bacteroidetes as the dominant taxa with nearly a quarter (61/259) of the LA communities studied. High Fusobacteria microbial communities were the most prevalent among LAs, but the relative abundances of Fusobacteria and Bacteroidetes were negatively correlated, and LA microbial communities rarely contained high abundances of both of these dominant phyla. Further, based on the presence of taxa discriminant of Bacteroidetes-dominated LAs within over 400 bovine gut communities, we provide evidence that Bacteroidetes-dominated abscess communities may originate in more distal portions of the bovine gut.

As tylosin reduced abscess prevalence but did not significantly impact abscess microbial communities, it follows that its mode of action is likely occurring in the gut rather than acting directly on LA microbial communities. However, there is currently no practical method for comprehensive antemortem investigation of LAs in cattle, and cattle in this study were harvested at single time point, at an age and stage of maturity that is typical for commercial beef production in North America. As such, it is not possible to determine when the infections associated with the abscesses that were investigated in this study were initiated, it is not known if the microbial community structure of LAs changes over time, and it is also unknown whether impacts of tylosin supplementation occur throughout the feeding period. A previous sequencing-based showed that tylosin can alter the microflora of the bovine gut, though neither *Fusobacterium* nor *Bacteroides* was impacted and the functional impact was minimal (Thomas et al., 2017), but culture-based studies have shown that the concentration of *Fusobacterium* in the rumen is lower in cattle fed tylosin (Nagaraja et al., 1999b). It will be important for future work investigating the impact of tylosin and antibiotic alternatives to focus on gut epithelium microbial communities and gut inflammation and permeability, as it seems this is where tylosin is acting to reduce liver abscess prevalence. Research in other species has established that microbial community composition can have a marked effect on gut inflammation and immunity (Schirmer et al., 2016; Buford, 2017) and could plausibly be the underlying mode of action of antimicrobial drugs in preventing LA occurrence in cattle. If tylosin or other interventions studied in these trials were directly impacting LAs by affecting growth and survivability of specific bacterial taxa (e.g., *Fusobacterium*, *Bacteroides*, *Trueperella*, *Porphyromonas*, or Enterobacteriaceae such as *Salmonella*), then we might expect to see differences in the relative abundance of these specific taxa among LA recovered from the different treatment groups. Our results, along with evidence that LA microbial communities may arise from sources throughout the GIT, together suggest that tylosin and other antibiotics may prevent LAs by acting indirectly on gut inflammation and permeability to prevent bacterial translocation into the portal system.

Here, we present the first examination of members of Bacteroidetes as the overwhelmingly dominant taxa within some LAs. While Fusobacteria-dominated communities were still by far the most common, Bacteroidetes-dominated communities represented almost a quarter (23%) of the abscesses sampled in this study. When compared across treatment groups in previous 16S sequencing studies, members of Bacteroidetes were the second or third most abundant phyla after Fusobacteria within abscesses (Weinroth et al., 2017; Amachawadi et al., 2021; Stotz et al., 2021) but have not previously been demonstrated as the dominant members of individual abscesses. Within these Bacteroidetes-dominated communities, the genera *Bacteroides* and *Porphyromonas* were the most abundant, species of which have previously been isolated from LAs. While they have largely been ignored as potential primary influencers of liver abscess syndrome in cattle, species of *Bacteroides* and *Porphyromonas* are commonly found in anaerobic infections and have been extensively implicated in non-liver abscess formation in humans (Gibson et al., 1998; Murakami et al., 2001; Van der Cruyssen et al., 2017). They also contain potent virulence factors and are capable of activating the inflammosome (Olsen and Yilmaz, 2016), making it feasible that they could significantly contribute to LA formation.

The pen-level prevalence of LAs was not associated with the proportion of abscesses dominated by Bacteroidetes or Fusobacteria. Treatment with tylosin or antibiotic alternatives had no significant impact on the proportion of either community type, suggesting they are impacting the formation of Fusobacteria and Bacteroidetes-dominated abscesses relatively equally. As *Fusobacterium*, *Bacteroides*, and *Porphyromonas* (the predominant genera in each LA community type) are all anaerobic, Gram-negative rods that have shown synergistic potential in non-cattle abscesses (Brook et al., 1984; Könönen et al., 2015), it follows that they would respond similarly to treatment. However, the slight decrease in the proportion of Bacteroidetes-dominated LAs in cattle given tylosin may warrant further investigation with a larger number of abscesses.

Aside from the abundant genera (i.e., *Fusobacterium*, *Bacteroides*, and *Porphyromonas*) within each community type, *Acinetobacter*, *Lactobacillus*, *Pseudomonas*, and *Psychrobacter* were discriminant of Fusobacteria-dominated communities and *Atopobium*, *Campylobacter*, *Helocococcus*, *Parvimonas*, and *Trueperella* were discriminant of Bacteroidetes-dominated abscesses. Except for *Lactobacillus*, discriminant taxa for both community types contain strains considered pathogenic. Members of *Acinetobacter*, *Campylobacter*, *Helocococcus*, *Parvimonas*, *Pseudomonas*, and *Psychrobacter* have all been associated with wound infections in the gut (Collins et al., 2004; Eliopoulos et al., 2008; Price et al., 2009; Okuda et al., 2010; Kaakoush et al., 2014), and despite their differential abundance between abscesses community type, their presence in LAs further emphasizes the importance of keeping a polymicrobial perspective when considering causes and potential prevention strategies for LA syndrome in cattle. *Trueperella* is commonly identified as a predominant microbial community member and considered a potential secondary etiological agent of bovine LAs (Reinhardt and Hubbert, 2015; Weinroth et al., 2017; Stotz et al., 2021), and its increased abundance in Bacteroidetes-dominated LAs is an important finding that emphasizes the importance of discriminating between community types in future research.

The hepatic portal vein drains blood from throughout the GI tract, from the rumen through the cranial rectum (Getty and Sisson, 1975; Clark et al., 2018), and bacterial translocation from the gut lumen into the portal blood provides a means for gut microbiota to seed the liver (Nagaraja and Lechtenberg, 2007). It is generally accepted that inflammation in the rumen as the result of *F. necrophorum*-associated ruminal acidosis provides gut pathogens access to the portal vein and transport to the liver, where *F. necrophorum* becomes the primary causative agent of abscess formation (Tadepalli et al., 2009). Lactate-producing organisms and *Lactobacillus* spp., specifically, are more abundant during ruminal acidosis (Nagaraja and Titgemeyer, 2007), and *Lactobacillus* being discriminant of Fusobacteria-dominated abscess communities supports the ruminal acidosis origin of LAs. However, since the portal vein drains the entire GIT, we hypothesized that the Bacteroidetes-dominated abscesses communities with low abundances of *Fusobacterium*, and potentially abscesses with more abundant Proteobacteria due to their elevated Enterobacteriaceae abundances, may have originated in a different segment of the gut. Indeed, *Bacteroides* and *Porphyromonas* were substantially more abundant in the large intestine then more proximal segments, and virtually absent from the rumen, omasum, and abomasum. Fusobacterium-discriminant taxa were more prevalent in more proximal segments (i.e., small intestine), albeit also virtually absent from the rumen, omasum, and abomasum. We acknowledge the limitations of investigating these liver community discriminant taxa in the gut, specifically the considerably low relative abundance and lack of absolute abundance values, but believe it provides preliminary evidence of liver abscess microbial communities originating in different portions of the bovine GIT. Future work investigating the origins of non-*Fusobacterium*-dominated liver abscess communities should include the absolute quantification of taxa of interest on the epithelium (e.g., *Fusobacterium*, *Lactobacillus*, *Bacteroides*, *Porphyromonas*, and *Trueperella*) at multiple locations of the GIT and include assays targeting gut inflammatory responses.

## Data Availability Statement

The datasets presented in this study can be found in online repositories. The names of the repository/repositories and accession number(s) can be found at: https://www.ncbi.nlm.nih.gov/, PRJNA472428; https://www.ncbi.nlm.nih.gov/, PRJNA804092.

## Ethics Statement

The animal study was reviewed and approved by Colorado State University Research Integrity and Compliance Review Office (Protocol number 102616). Written informed consent was obtained from the owners for the participation of their animals in this study.

## Author Contributions

CW, KH, TB, JM, KB, and PM participated in and provided oversight and participated in the experimental design, laboratory procedures, report preparation, and securing funding. LP performed the data analysis and wrote the manuscript. All authors contributed to the article and approved the submitted version.

## Funding

This work was funded by the Beef Checkoff, Diamond V, DSM Nutritional Products, Colorado State University, and Texas A&M University.

## Conflict of Interest

TB was employed by the company Five Rivers Cattle Feeding.

The remaining authors declare that the research was conducted in the absence of any commercial or financial relationships that could be construed as a potential conflict of interest.

## Publisher’s Note

All claims expressed in this article are solely those of the authors and do not necessarily represent those of their affiliated organizations, or those of the publisher, the editors and the reviewers. Any product that may be evaluated in this article, or claim that may be made by its manufacturer, is not guaranteed or endorsed by the publisher.

## Footnotes

^1^ https://huttenhower.sph.harvard.edu/galaxy

## Supplementary Material

The Supplementary Material for this article can be found online at: https://www.frontiersin.org/articles/10.3389/fmicb. 2022.882419/full#supplementary-material

Supplementary Figure S1 Bar plots demonstrating differences in the proportion LA communities classified as high Bacteroidetes and high Fusobacteria in pens with low, middle, or high LA prevalence rates in across both trials. Error bars represent the standard error of the mean, and no significant differences were detected (Pairwise Wilcoxon rank-sum with Benjamini–Hochberg correction, *p* > 0.05, *n* = 21–22).

Supplementary Figure S2 Bar plots demonstrating differences in the proportion LA communities classified as high Bacteroidetes and high Fusobacteria in pens containing animals given the control diet, diet supplemented with SCFP, diet supplemented with EO, or diet supplemented with tylosin. Error bars represent the standard error of the mean, and no significant differences were detected (Pairwise Wilcoxon rank-sum with Benjamini–Hochberg correction, *p* > 0.05, *n* = 9–24).
