# Supplementary figures and images for: Not All Liver Abscesses Are Created Equal: The Impact of Tylosin and Antibiotic Alternatives on Bovine Liver Abscess Microbial Communities and a First Look at Bacteroidetes-Dominated Communities
Source: Front Microbiol. 2022 Apr 27;13:882419. doi: 10.3389/fmicb.2022.882419 (PMC9094069; doi:10.3389/fmicb.2022.882419)

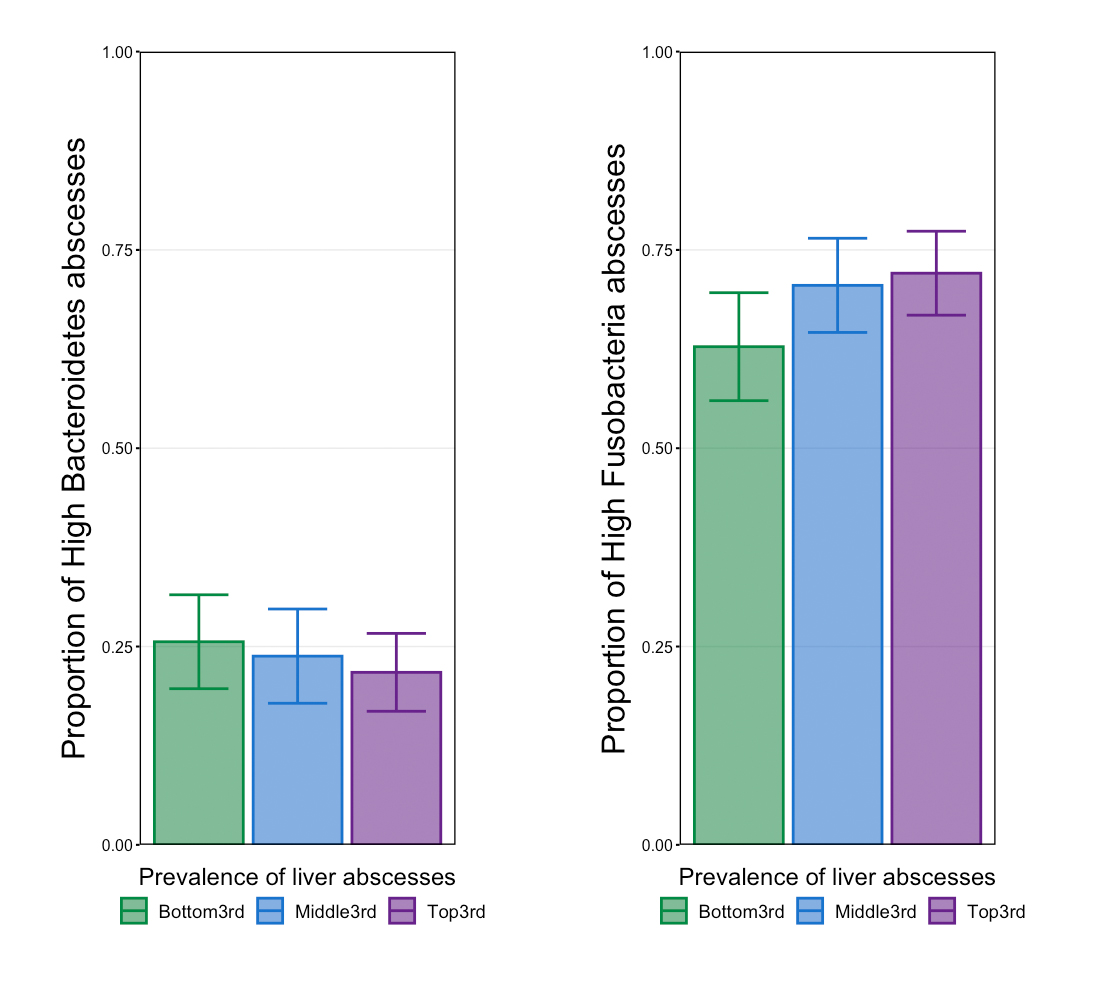

Supplement: Supplementary Figure S1 — Bar plots demonstrating differences in the proportion LA communities classified as high Bacteroidetes and high Fusobacteria in pens with low, middle, or high LA prevalence rates in across both trials. Error bars represent the standard error of the mean, and no significant differences were detected (Pairwise Wilcoxon rank-sum with Benjamini–Hochberg correction, p > 0.05, n = 21–22). [file Image_1.JPEG]

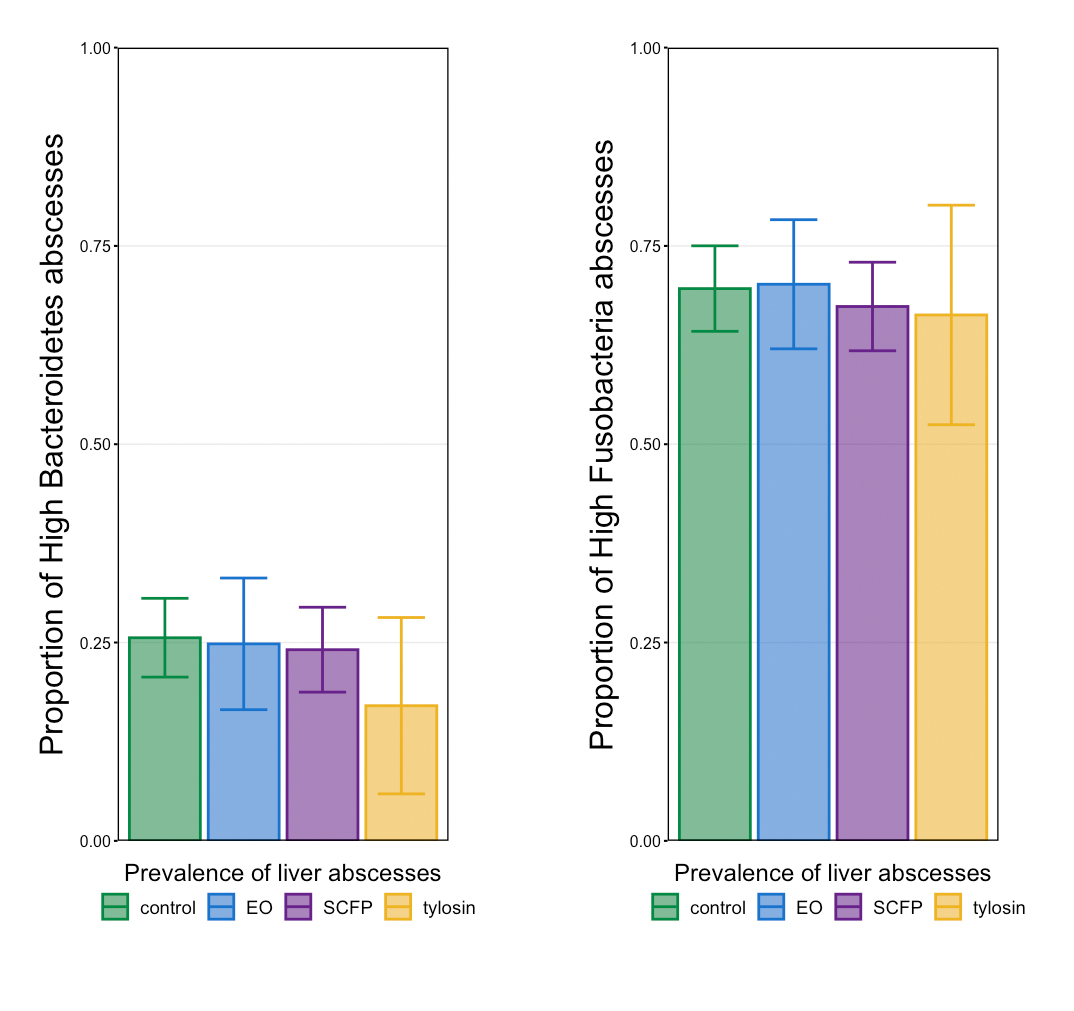

Supplement: Supplementary Figure S2 — Bar plots demonstrating differences in the proportion LA communities classified as high Bacteroidetes and high Fusobacteria in pens containing animals given the control diet, diet supplemented with SCFP, diet supplemented with EO, or diet supplemented with tylosin. Error bars represent the standard error of the mean, and no significant differences were detected (Pairwise Wilcoxon rank-sum with Benjamini–Hochberg correction, p > 0.05, n = 9–24). [file Image_2.JPEG]
