# Supplementary material for: Not All Liver Abscesses Are Created Equal: The Impact of Tylosin and Antibiotic Alternatives on Bovine Liver Abscess Microbial Communities and a First Look at Bacteroidetes-Dominated Communities
Source: Front Microbiol. 2022 Apr 27;13:882419. doi: 10.3389/fmicb.2022.882419 (PMC9094069; doi:10.3389/fmicb.2022.882419)
Supplement: Supplementary file 3 [file Data_Sheet_1.zip › Table S1.docx]

**Table S1.** Proportion of 16S rRNA gene sequence amplicon sequence variants (ASVs) that were classified at each taxonomic rank.

|  | **Taxonomic rank** | | | | |
| --- | --- | --- | --- | --- | --- |
|  | Phylum | Class | Order | Family | Genus |
| ASVs classified | 99.8% | 99.7% | 99.5% | 98.6% | 96.1% |
