# Supplementary material for: Not All Liver Abscesses Are Created Equal: The Impact of Tylosin and Antibiotic Alternatives on Bovine Liver Abscess Microbial Communities and a First Look at Bacteroidetes-Dominated Communities
Source: Front Microbiol. 2022 Apr 27;13:882419. doi: 10.3389/fmicb.2022.882419 (PMC9094069; doi:10.3389/fmicb.2022.882419)
Supplement: Supplementary file 3 [file Data_Sheet_1.zip › Table S2.docx]

**Table S2**. PERMANOVA and PERMDISP results from comparisons between microbial communities associated with different LA community types (high Bacteroidetes, high Fusobacteria, high Proteobacteria, high Firmicutes, or other) based on generalized UniFrac values. Significant results are bolded (p-adj. < 0.05).

| **Test** | **Df** | **SS** | **Pseudo-F** | **R^2^** | **p-adj.** | **PERMDISP (p-adj.)** |
| --- | --- | --- | --- | --- | --- | --- |
| Fusobacteria v. Bacteroidetes | 1 | 1.279 | 47.024 | 0.164 | **0.0002** | 0.2293 |
| Fusobacteria v. Proteobacteria | 1 | 0.552 | 17.493 | 0.085 | **0.0002** | **0.0001** |
| Fusobacteria v. Firmicutes | 1 | 0.324 | 11.076 | 0.057 | **0.0002** | **0.0114** |
| Fusobacteria v. other | 1 | 0.265 | 9.202 | 0.048 | **0.0002** | 0.0788 |
| Bacteroidetes v. Proteobacteria | 1 | 0.623 | 18.236 | 0.211 | **0.0002** | **0.0001** |
| Bacteroidetes v. Firmicutes | 1 | 0.408 | 14.729 | 0.187 | **0.0002** | **0.0070** |
| Bacteroidetes v. other | 1 | 0.355 | 13.477 | 0.176 | **0.0003** | **0.0430** |
| Proteobacteria v. Firmicutes | 1 | 0.098 | 1.000 | 0.077 | 0.509 | 0.0898 |
| Proteobacteria v. other | 1 | 0.092 | 0.949 | 0.079 | 0.516 | **0.0262** |
| Firmicutes v. other | 1 | 0.068 | 0.929 | 0.117 | 0.553 | 0.4484 |

Abbreviations: v., versus; Df, degrees of freedom; SS, sum of squares; p-adj., adjusted p-value
