# Supplementary material for: Not All Liver Abscesses Are Created Equal: The Impact of Tylosin and Antibiotic Alternatives on Bovine Liver Abscess Microbial Communities and a First Look at Bacteroidetes-Dominated Communities
Source: Front Microbiol. 2022 Apr 27;13:882419. doi: 10.3389/fmicb.2022.882419 (PMC9094069; doi:10.3389/fmicb.2022.882419)
Supplement: Supplementary file 3 [file Data_Sheet_1.zip › Table S3.docx]

| **high Bacteroidetes** | **high Fusobacteria** | **high Proteobacteria** | **high Firmicutes** | **Other** |
| --- | --- | --- | --- | --- |
| *Bacteroides*  51.27 ± 2.67 | *Fusobacterium*  92.22 ± 0.48 | *Fusobacterium*  41.61 ± 7.80 | *Fusobacterium*  57.36 ± 2.61 | *Fusobacterium*  66.95 ± 2.08 |
| *Fusobacterium*  36.06 ± 2.03 | *Bacteroides*  1.74 ± 0.23 | *Agrobacterium*  6.61 ± 6.56 | un. Veillonellaceae  5.49 ± 1.33 | *Bacteroides*  4.93 ± 1.81 |
| *Porphyromonas*  4.91 ± 1.37 | un. Fusobacteriaceae  0.49 ± 0.06 | *Halomonas*  2.85 ± 1.53 | *Bacteroides*  2.86 ± 0.68 | un. Enterobacteriaceae  2.28 ± 0.87 |
| *Campylobacter*  2.61 ± 0.78 | un. Enterobacteriaceae  0.31 ± 0.03 | *Campylobacter*  2.21 ± 2.14 | un. Ruminococcaceae  2.77 ± 1.18 | *Halomonas*  1.69 ± 0.39 |
| un. Bacteroidales  1.01 ± 0.35 | *Lactobacillus*  0.29 ± 0.04 | *Bacteroides*  2.20 ± 0.55 | un. Clostridiales  1.76 ± 0.30 | un. Veillonellaceae  1.49 ± 0.79 |
| *Trueperella*  0.30 ± 0.10 | *Acinetobacter*  0.27 ± 0.04 | *Acinetobacter*  2.14 ± 0.92 | un. Bacteria  1.49 ± 0.33 | un. Fusobacteriaceae  1.48 ± 0.21 |
| un. Veillonellaceae  0.28 ± 0.08 | *Halomonas*  0.24 ± 0.05 | un. Enterobacteriaceae  2.06 ± 0.65 | un. Enterobacteriaceae  1.33 ± 0.12 | *Corynebacterium*  1.24 ± 0.68 |
| un. Fusobacteriaceae  0.28 ± 0.06 | un. Bacteria  0.22 ± 0.04 | *Lactobacillus*  1.57 ± 0.52 | *Sporomusa*  1.24 ± 0.28 | *Shewenella*  0.66 ± 0.08 |
| *Filifactor*  0.23 ± 0.10 | un. Veillonellaceae  0.16 ± 0.05 | un. Iii1-15 (Acidobacteria)  1.51 ± 1.16 | *Halomonas*  1.12 ± 0.15 | *Blautia*  0.63 ± 0.34 |
| un. Enterobacteriaceae  0.13 ± 0.03 | *Streptococcus*  0.12 ± 0.02 | un. Veillonellaceae  1.38 ± 0.78 | *Treponema*  1.03 ± 0.45 | un. Iii1-15 (Acidobacteria)  0.56 ± 0.43 |

**Table S3.** Mean relative abundance plus or minus the standard error of the mean for the ten most abundant taxonomic genera within each LA microbial community type: high Bacteroidetes (n=180), high Fusobacteria (n=61), high Proteobacteria (n=9), high Firmicutes (n=5), other (n=4).

Abbreviations: un., unclassified
